# Complete genome sequence of *Rhodococcus globerulus* AX7B, an abscisic acid-metabolizing strain

**DOI:** 10.1128/mra.00685-25

**Published:** 2025-09-08

**Authors:** A. A. Belimov, P. V. Guro, E. A. Sekste, A. L. Sazanova, T. S. Azarova, A. I. Shaposhnikov, O. S. Yuzikhin, V. Y. Shahnazarova, N. A. Vishnevskaya, M. I. Lebedinsky, V. I. Safronova

**Affiliations:** 1 All-Russia Research Institute for Agricultural Microbiology (ARRIAM) 117473, St.Petersburg, Russia; Indiana University Bloomington, Bloomington, Indiana, USA

**Keywords:** abscisic acid, rhizobacteria

## Abstract

We report the genome sequence of *Rhodococcus globerulus* strain AX7B, isolated from the pea rhizosphere and capable of utilizing abscisic acid as a sole carbon source. The complete genome consists of a 6.62 Mb chromosome and a 0.44 Mb plasmid, containing 6,344 protein-coding genes and 70 RNA genes.

## ANNOUNCEMENT

Abscisic acid (ABA) is a key phytohormone in plant stress responses. Although ABA production by rhizobacteria is well known, its microbial degradation is less understood (1). Early studies identified *Corynebacterium, Rhodococcus, Novosphingobium*, and *Rhodococcus qingshengii* as ABA degraders, with the latter enhancing heavy metal phytoremediation (2–4). Rapid ABA degradation by soil microbes suggests diverse catabolic pathways. Due to ABA’s ecological and biotechnological significance, studying ABA-metabolizing bacteria is critical. Strain AX7B of *Rhodococcus globerulus,* that is capable of utilizing ABA as a carbon source was isolated from pea (*Pisum sativum* L.) rhizosphere in Veliky Novgorod, Russia (57.148279°N, 31.179109°E) on selective agar medium with 0.25 mg ABA mL^−1^ as the sole carbon source, containing (g L^−1^): NH_4_NO_3_ – 0.3, KH_2_PO_4_ – 0.4, K_2_HPO_4_ – 2.0, MgSO_4_ ×7H2O – 0.2, CaCl_2_ – 0.1, FeSO_4_ – 0.005, H_3_BO_3_ – 0.002, ZnSO_4_ – 5, Na_2_MoO_4_ – 1, MnSO_4_ – 3, CoSO_4_ – 1, CuSO_4_ – 1, NiSO_4_ – 1, agar – 15, pH =6.5 (3). Colonies appeared after three days at 28°C. A single colony was re-cloned twice to obtain a pure culture. Genomic DNA was isolated using the DNeasy Blood & Tissue KIT (Qiagen, Germany) according to the manufacturer’s recommendations from a pure culture grown in R2A broth at 28°C for 48 hours. The library was prepared with ONT’s Native Barcoding Kit (SQK-NBD114.24) without fragmentation or size selection. Sequencing was performed using a MinION R10 flow cell (ONT, UK). Basecalling was performed using Dorado (v0.7.3, https://github.com/nanoporetech/dorado). Quality control of the raw reads was provided by NanoStat (v1.6.0) (5). Sequencing yielded 88,000 reads (7,861 bp avg length; N50=14,193 bp; Q =12.9). The ONT reads were filtered using Filtlong (v0.2.1, https://github.com/rrwick/Filtlong), with options: --min_length 1000; --keep_percent 95, resulting in 67,002 reads and an average length of 9,808 bp. Flye v2.9 assembly yielded two contigs: a circular chromosome without a specific start point, and a linear plasmid (6). Chromosome terminal overlaps were checked via NUCmer v4.0.0rc1 self-alignment (7). Consensus sequences were generated by polishing the assembly using Medaka (v1.8.0; https://github.com/nanoporetech/medaka) and the raw reads. Genomic statistics were measured using QUAST (v5.0.2) (8). The total size of the assembly is 7.1 Mbp (a chromosome of 6,623,570 bp and a plasmid of 438,797 bp), N_50_ value is 6,623,570  bp with an average coverage of 90× and a GC content of 61.65%. Default parameters were used for all software unless otherwise noted. Annotation of the assembly using PGAP (v6.10) resulted in 6,344 protein-coding genes and 70 RNA genes (15 rRNAs, 52 tRNAs, and 3 ncRNAs) (9). Initial taxonomic identification via 16S rRNA gene sequencing (PX069515) compared with type strains by BLASTn comparison revealed 99.79% similarity to *Nocardia globerula* DSM 44596 (NR_104795.1) and 99.46% to *R. globerulus* DSM 43954 (NR_026184.1) (10). Following genus identification, average nucleotide identity (ANI) analysis was performed against *R. globerulus* NBRC 14531 (GCF_001894805.1) using FastANI v1.33 and dDDH via TYGS (https://tygs.dsmz.de) (11). Results showed 98.75% ANI and 89.3% dDDH, supporting classification of strain AX7B as *R. globerulus*.

## Data Availability

All data are available at GenBank under BioProject accession number PRJNA1218496. The BioSample accession number is SAMN46524525. The raw MinION data can be found under number SRR32263048 and the assembly accession number CP180408.1-CP180407.1.
